# Neurodegeneration in Alzheimer Disease: Role of Amyloid Precursor Protein and Presenilin 1 Intracellular Signaling

**DOI:** 10.1155/2012/187297

**Published:** 2012-02-08

**Authors:** Mario Nizzari, Stefano Thellung, Alessandro Corsaro, Valentina Villa, Aldo Pagano, Carola Porcile, Claudio Russo, Tullio Florio

**Affiliations:** ^1^Section of Pharmacology, Department of Internal Medicine and Center of Excellence for Biomedical Research, University of Genova, 16132 Genova, Italy; ^2^IRCCS Azienda Ospedaliera Universitaria San Martino-Istituto Nazionale per la Ricerca sul Cancro (IST), Università di Genova, 16132 Genova, Italy; ^3^Department of Health Sciences, University of Molise, 86100 Campobasso, Italy

## Abstract

Alzheimer disease (AD) is a heterogeneous neurodegenerative disorder characterized by (1) progressive loss of synapses and neurons, (2) intracellular neurofibrillary tangles, composed of hyperphosphorylated Tau protein, and (3) amyloid plaques. Genetically, AD is linked to mutations in few proteins amyloid precursor protein (APP) and presenilin 1 and 2 (PS1 and PS2). The molecular mechanisms underlying neurodegeneration in AD as well as the physiological function of APP are not yet known. A recent theory has proposed that APP and PS1 modulate intracellular signals to induce cell-cycle abnormalities responsible for neuronal death and possibly amyloid deposition. This hypothesis is supported by the presence of a complex network of proteins, clearly involved in the regulation of signal transduction mechanisms that interact with both APP and PS1. In this review we discuss the significance of novel finding related to cell-signaling events modulated by APP and PS1 in the development of neurodegeneration.

## 1. Introduction

Alzheimer disease (AD) is a neurodegenerative disease clinically characterized by progressive dementia, and, neuropathologically, by loss of synapses and neurons, gliosis, and the presence of both amyloid plaques and neurofibrillary tangles. The main amyloid components of plaques are a family of short peptides (A*β*) of 40 or 42 amino acids, in the most common forms, derived from the proteolysis of the type I protein, amyloid *β* precursor protein *(A*β*PP)*, upon sequential cleavage by *β*- and *γ*-secretases [1]. *γ*-secretase has been characterized as a multiprotein complex in which presenilins 1 and 2 have a regulatory role [1]. Familial AD forms (FADs) are caused by the overexpression or by mutations in the *A*β*PP* gene, or by mutations on the presenilins (*presenilins 1 and 2*). 

The molecular mechanisms underlying the development of AD are not yet known, and also the physiological role of A*β*PP is still unclear [2].

In particular, it is still debated whether presenilins (PSs) familial mutations cause gain or loss of function in the *γ*-secretase complex. PS mutations have been presumed to cause FAD by enhancing production of the more toxic A*β*42 over the A*β*40 isoform, thereby conferring a toxic gain of function [3]. However, a number of recent studies have shown that clinically relevant PS mutations impair A*β*40 production without affecting A*β*42 production, leading to the revised view that pathogenic PS mutations consistently shift the cleavage specificity of the mutant protein to favor production of A*β*42 at the cost of A*β*40 [4, 5]. On the other hand, it has been recently suggested that, at least, some FAD-associated PS mutations can cause a nearly complete loss of the mutant protein's ability to support *γ*-secretase activity rather than an absolute or relative overproduction of A*β*42 [6]. Hence, a loss of function can be associated to a shift of the cleavage specificity (A*β*42 at the cost of A*β*40), or to another unknown target substrate of the *γ*-secretase activity. In this case, the loss of PS function may be a primary event, in the adult mammalian brain, triggering a putative pathogenic cascade which leads to neurodegeneration in AD [7].

Several studies suggest a relevant role for A*β*PP in maintaining active synapses, and recent evidence has indicated the presence of A*β*PP in the postsynaptic density, where it may interact with NMDA receptors, thus supporting the observation that NMDA receptors regulated trafficking and processing of A*β*PP, although *via* a controversial mechanism of action [8].

**Figure 1:**
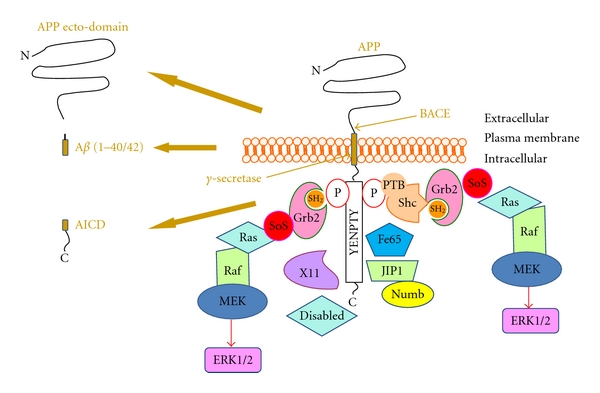
Schematic representation of A*β*PP processing, the adaptor proteins interacting with its intracellular domain and the pathway leading to ERK1/2 activation. In the left panels is reported the transmembrane protein APP, before and after ITS sequential beta secretase (BACE) and gamma secretase cleavage, with its final products, AICD, APP ectodomain, and beta amyloid peptide (1–40/1–42). In the right part of the figure are indicated the protein interacting with APP intracellular domain, upon or independently from tyrosine phosphorylation. The adaptor proteins Shc and Grb2 through their phosphotyrosine-binding domain (PTB) and src homology domain (SH2) are able to directly bind tyrosine-phosphorylated APP, resulting in the recruitment of the components of the MAP kinase cascade (SoS, ras, Raf, MEK) leading to ERK1/2 activation. Grb2 may participate in this pathway either by direct binding to APP or being recruited by Shc. Alteration in ERK1/2 activity induced in this way may contribute to neurodegeneration in AD. Transduction pathway adaptors (X11, disabled, Fe65, JIP1, and Numb) that bind APP in the absence of tyrosine phosphorylation depicted are also shown.

**Figure 2:**
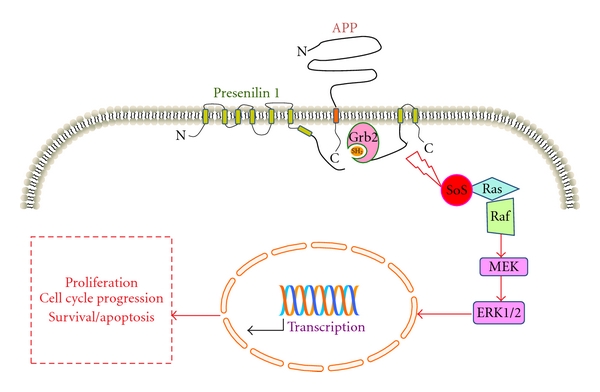
Schematic representation of the intracellular pathway by which A*β*PP and PS1 control the activation of the MAPK/ERK1/2 cascade and their final biological effects. In the figure is specified the interaction between APP intracellular domain and PS1 C-terminus, with the adaptor protein Grb2. Grb2 can bind simultaneously to APP and PS1 (as measured in FRET experiments) leading to the MAPK ERK1/2 cascade activation. In AD an aberrant activation of ERK1/2 induced by APP and/or PS1 can determine the tentative activation of the cell cycle that, in postmitotic neurons, may induce cells to undergo apoptosis.

**Figure 3:**
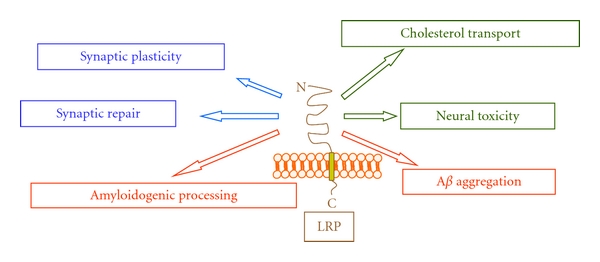
Role of LPR8 activation in normal brain functioning and in neurodegeneration during AD. In the figure are indicated the different roles in which LRP8 transmembrane protein is involved, in the healthy brain and AD pathogenesis.

Moreover, recent findings have also suggested that A*β*PP, through an NPTY motif located in its cytodomain, and PSs form functional complexes with different signaling protein, supporting the hypothesis that A*β*PP and PS1 are at the centre of a complex network of interactions, likely involved in multiple cell-signaling events which are still unknown (Figures 1 and 2) [9, 10]. Even apolipoprotein E (ApoE), which is the most relevant risk factor for developing late-onset AD, rather than being a mere A*β* chaperone, might be involved in complex-signaling pathways through its multiple receptors (LRPs), such as those bearing to the low-density lipoprotein receptor family (LDLR) (for review see [11]). LRPs participate in neuronal functions modulating neurotransmission and thus synaptic stability [12], and several data indicate that LRPs could modulate A*β*PP processing through the regulation of its endocytic trafficking, implying a possible association between LRPs activity and AD onset (Figure 3) [13]. Taken together, these data suggest a model that links the functions of A*β*PP, PSs, and LRPs in physiological and pathophysiological conditions as relevant actors in neuronal intracellular signaling.

This review will focus on the involvement of A*β*PP in cell signaling, exploring the possibility that posttranslational modifications on its C-terminal domain may modulate, together with PSs and LRPs, intracellular pathways involved in cell-cycle progression that in postmitotic neurons may induce neurodegeneration.

## 2. APP Processing, Interacting Proteins, and Intracellular Signalling

The main amyloid components of senile plaques result from the proteolytic processing of A*β*PP by *β*-secretase (BACE1), leading to the formation of C-terminal fragments (CTFs) that are subsequently cleaved by the “*γ*-secretase-complex” which is responsible for the formation of A*β* (40 or 42 amino acids in length) and the A*β*PP intracellular domain peptide (AICD) of 58 or 56 amino acids (Figure 1) [1].

These amyloid peptides are considered mainly responsible for the neurodegeneration that occurs in AD. Thus, the “amyloid hypothesis” sustains that the first step during AD development is the accumulation and the subsequent deposition of A*β* peptides [1, 2, 10, 14–23].

The generation of A*β*40/42 peptides, by the sequential proteolytic activity of *β*- and *γ*-secretases, is enhanced by mutations in A*β*PP and PSs, and it may be prevented by the action of a third protease, the *α*-secretase, that cleaves A*β*PP within the A*β* region, thus resulting in the formation of a different subset of CTFs (*α*-CTFs) that upon *γ*-cleavage generate shorter and nonamyloidogenic fragments [24].

However, from the point of view of the signaling activity of CTFs, it is still unclear whether *α*- and *β*-stubs or the AICD fragments generated by *γ*-secretase might represent protective or pathologically related molecule [25].

As far as AICD fragments are concern, it was reported that, after binding Fe65 (Figure 1), an adaptor protein mediating assembly of multimolecular complexes through a variety of protein-interaction domains, and the histone acetyltransferase Tip60, AICD translocate into the nucleus where it acts as gene transcription regulators [24, 26–29]. However, this latter event is still debated, because AICD displays a very short half-life and a poorly characterized* in vivo* transcriptional activity [30].

Recent data have demonstrated that A*β*PP may signal to the nucleus also using a *β*-secretase-independent mechanism that involves membrane sequestration and phosphorylation of Tip60 [31].

More recently, Stante et al. have suggested that the presence of Fe65 into the nucleus may have a protective role, and that its translocation depends on A*β*PP. They propose that DNA repair defects could significantly contribute to the neurodysfunction and neurodegeneration observed in AD, and that an involvement of the Fe65-APP complex in the response of the cells to DNA damage and in the DNA repair machinery could represent a possible mechanism contributing to neuronal degeneration observed in AD pathology [32].

Indeed, new data suggest that, during embryonic development, AICD release, triggered by extracellular signals activating the *β*-secretase-dependent cleavage, may be involved in the control of neurogenesis [33]. Conversely, Vogt and Coll showed that the overexpression of AICD in mice caused abnormal neuronal networks and increased seizure susceptibility [34]. Other studies demonstrated that AICD may induce the expression of neprilysin, an enzyme known for its specific A*β*-degrading activity, through a direct modulation of its promoter [35].

At the same time, it is noteworthy that the C-terminal portion of A*β*PP and in particular the last 20 amino acids in the cytoplasmic tail which contains the well-known YENPTY (Figure 1) motif present in several receptor tyrosine kinase (TK) is a docking site for different intracellular proteins involved in signal transduction. Traditionally, this sequence was described as internalization motif, while now it has been recognized to play a central role also in the regulation of multiple interactions with intracellular proteins [9, 36]. In particular, in receptor TK, tyrosine residue can be phosphorylated to generate the NPXpY motif, which represents a docking site for several intracellular adaptor proteins through the phosphortyrosine-binding domain (PTB). Similarly, the adaptor proteins Shc and Grb2 can bind A*β*PP (or its CTFs) in the presence of phosphorylated tyrosine in this motif (Figure 1). However, A*β*PP (or its CTFs) and the A*β*PP-related proteins, APLP1 and APLP2, can also interact with several other signalling proteins, including X11 [37], Fe65 [37, 38], mDab [39], c-Abl [40], JIP-1 [41], and Numb [36, 41], (Figure 1) independently of the phosphorylation of the tyrosine residue within the YENPTY motif. From a functional point of view, the interaction between the neuron-specific adaptor protein Fe65 and A*β*PP via the second PTB domain of Fe65 [37, 38] was shown to modulate A*β*PP processing, favoring the generation of A*β* and A*β*PP trafficking, in several cell lines [26, 42]. Another adaptor that binds to A*β*PP is mDAB. It is a protein related to the reelin pathway and interacting with YENPTY motif through a PTB domain. mDAB is active during embryogenesis, where it regulates the position of neurons in the brain laminar structure [43], and mDAB binding increases the amounts of mature A*β*PP and A*β* formation [44]. On the contrary, X11 stabilizes A*β*PP conformation in membrane, inhibiting A*β* secretion in cultured cells [45], likely impairing A*β*PP trafficking to sites containing active *γ*-secretase complexes [46]. JIP's are member of JNK-scaffolding family proteins kinases, implicated in different signal pathway, including neuronal apoptosis. JNK-interacting proteins JIP1b and JIP2 bind to the cytoplasmic tail of A*β*PP. The expression of JIP1b stabilizes immature A*β*PP and decreases the A*β*PP ectodomain, A*β*40/*β*42 and CTFs abundance [47].

All these observations suggest that some of these protein-protein interactions may play a role in the modulation of the amyloidogenic pathway and thus might have a role in neurodegeneration.

The role of A*β* peptides as unique cause of neuronal toxicity and AD is highly debated, and recent data have challenged the “amyloid only” hypothesis, questioning the role of APP and PSs as mere amyloid productors. The central role of APP and PSs in the genesis of AD is unquestionable; however, phenotypical heterogeneity among patients, and even among familial patients with the same genetic mutation, is commonly observed, implying that other genes might have a role in regulating the onset and severity of the neurodegeneration in FAD and, likely, in sporadic AD. For these reasons, considering that APP and PSs are key players in a complex network of interactions with many different intracellular adaptors, it is tempting to hypothesize that, in parallel to amyloid formation, APP and PSs may induce neurodegeneration through specific alterations in neuronal signaling pathways [48].

In this context, it was reported that other two adaptor proteins, which have been involved in the regulation of the amyloidogenic pathway, ShcA and growth factor receptor-bound protein 2 (Grb2) are able to interact with the cytodomain of A*β*PP in the presence of specific tyrosine 682 phosphorylation in the YENPTY motif of A*β*PP cytodomain [36, 49]. ShcA (or ShcC) adaptors connect growth factor receptors to specific signaling pathways (typically Ras/ERK1/2 pathway but also PI3K/Akt signalling) and are involved in cell proliferation differentiation and apoptosis and neuronal development [50, 51]. Also the role of Grb2 in Ras-signaling pathway is well known as well as its involvement in the activation of the mitogen-activated protein kinase (MAPK) pathways cascade (Figures 1 and 2) [50, 52–54]. It is worth noting that ERK1/2 activity is increased in AD brains [55–57] and that activated MAPKs have been involved in the abnormal hyperphosphorylation of Tau in AD [58].

The pathogenic correlation between Shc/Grb2 binding to A*β*PP during AD development is supported by the observation that the complexes A*β*PP (or CTFs)/ShcA or Grb2 are significantly increased in AD brain as compared to controls [55]. The increased phosphorylation/activation of ERK1/2, often described in AD brain, is also observed in thrombin-activated astrocytes [55], suggesting that, in this model, ERK1/2 may be activated by A*β*PP through ShcA. These data give prominence to the biological importance of A*β*PP phosphorylation for its functions and the regulation of intracellular adaptor binding as events responsible for the induction of glial-associated mitogenic pathway. Furthermore, ERK1/2, activated by A*βin vitro*, plays a role in A*β*PP processing and phosphorylates Tau in a PHF-Tau similar manner [59]. However, it is conceivable that a different signaling A*β*-independent might as well activate tau phosphorylation by ERK1/2 via the intracellular signaling regulated by the A*β*PP/CTFs-Shc-Grb2 pathway (Figure 1).

A*β*PP cytodomain also interacts with other proteins directly linked to signal transduction mechanisms. In particular, A*β*PP binds to the heterotrimeric GTP-binding protein Go [60–63] that comprises up to 1% of all membrane-associated proteins in the developing nervous system [55]. There is evidence that A*β*PP cytodomain binds proteins involved in cell-cycle regulation such as A*β*PP-binding protein 1 (APP-BP1) [64] and p-21-activated kinase 3 (PAK3) [65] which is a serine/threonine kinase involved in DNA synthesis and neuronal apoptosis. These data are consistent with a model in which A*β*PP is a component of a Go multiprotein complex, including PAK3, to transduce extracellular signals to the cytoplasm. In this model, the FAD APP-mediated pathway, leading to tentative neuronal cell-cycle activation (see below), consists of the APP-Go-PAK3 formation, followed by the activation of the A*β*PP-BP1 through JNK [25].

Considering all these aspects, it is possible to hypothesize that posttranslational modifications of A*β*PP, or in its CTFs, such as a selective phosphorylation, might couple them, to different cellular pathways. These observation supports the hypothesis that A*β*PP may act as a receptor/transducer molecule in multiple cell-signaling events, the comprehension of which may have implications either for the normal biological function of A*β*PP, for its processing and for its pathological role in the genesis of AD [66–68].

## 3. Presenilins Modulation of Intracellular Signaling

Presenilins 1 and 2 are multitransmembrane proteins that, associated to nicastrin, APH-1 and PEN-2, form high-molecular *γ*-secretase complex, involved in A*β* production *via* intramembrane cleavage of A*β*PP (Figures 1 and 2) [69–71]. These proteins are highly expressed in brain but have been detected also in several different tissues. Amount of PSs are localized in the nuclear membrane, kinetochores, and centrosomes [72, 73]. At present more than 182 different mutations (and some deletions) in PS1 have been associated with inherited early onset AD (Alzheimer disease and Frontotemporal Dementia Mutation Database 2006) [56, 74, 75] while only 13 mutations have been found in PS2 that are definitively linked to FAD [15, 16, 76].

Besides their involvement in A*β* formation, PSs regulate the cleavage of other signaling receptors and transducers such as Notch-1, ErbB4, DC44, and LDL-receptor-related proteins and cadherins [1, 69, 77–79]. PSs also affect different other signaling molecules, such as wingless-type MMTV integration site family (Wnt) signal transduction pathway, which is evolutionary conserved and controls many events during the embryogenesis [80]. At cellular level, this pathway regulates morphology, proliferation, and motility of the cell. Wnt pathway plays a central role during tumorigenesis, and the inappropriate activation of this pathway has been observed in several human cancers [81]. It has been shown that Wnt-ligand-mediated signaling leads to the accumulation of cytosolic *β*-catenin. Cytosolic *β*-catenin will then translocate into the nucleus to bind to members of the T-cell factor (Tcf)/lymphoid-enhancing factor (Lef) family of DNA-binding proteins leading to the transcription of Wnt target genes. In the absence of Wnt ligand, axin recruits CK1 causing the initiation of the *β*-catenin phosphorylation cascade by glycogen synthase kinase-3 *β* (GSK-3*β*). Phosphorylated *β*-catenin is recognized by *β*-transducin repeat-containing protein (*β*-TrCP) and degraded by the proteosome, reducing the level of cytosolic *β*-catenin. It was reported that *β*-catenin interacts with PSs, and that PS1 promotes *β*-catenin degradation regulating phosphorylation by cyclin-dependent kinase 5 (CDK5) and GSK-3*β* [82–84]. Importantly, GSK-3*β* was implicated in various neurological disorders, including AD [85]. Gosal and Coll showed that AICD-overexpressing transgenic mice may have an abnormal activation of GSK-3*β*. These mice exhibit AD-like characteristics, including hyperphosphorylation and aggregation of tau, neurodegeneration, and working memory deficits that are prevented by treatment with lithium [86].

In cultured cells expressing PSs FAD mutants, the intracellular trafficking of *β*-catenin is altered, while in cells from PS-null animals cytosolic *β*-catenin levels and *β*-catenin-mediated Lef/Tcf signaling are increased [83], thus resulting in the activation of the downstream target cyclin D1 and accelerated entry into the S phase of the cell cycle [87].

Another relevant role for PSs is Notch processing. Notch signaling is involved in cell fate regulation, cell differentiation, proliferation, and apoptosis as well as neurodegeneration [88, 89]. Notch is a membrane receptor whose C-terminal domain (NICD), upon interaction with appropriate ligands, translocates into the nucleus where it activates the CSL family of transcription factors. NICD formation depends on *γ*-secretase complex as the AICD fragment of A*β*PP [78].

PSs play a role in apoptosis, since FAD mutants cause cell death or induce secondary events that may lead to apoptosis [90]. Animals, in which PS1 and PS2 genes are deleted, show deficit in learning, memory, synaptic function and neuronal death [91]. The processes beneath these effects are unknown, but the findings that PS1 interacts with antiapoptotic member of Bcl-2 family might indicate a possible mechanism [92, 93].

PS1 is also essential for efficient N-cadherin trafficking from ER to plasma membrane. Cadherins, including E-cadherin and neuronal cadherin (N-cadherin), are a family of type I transmembrane proteins that mediate Ca^2+^-dependent cell-cell adhesion, and recognition [94, 95]. PS1-mediated delivery of N-cadherin to the plasma membrane is important to exert its physiological function, including the control of the state of cell-cell contact [96].

PS1 is involved in the intramembrane cleavage of CD44, a cell surface adhesion molecule for the extracellular matrix components which is implicated in a wide variety of physiological and pathological processes including the regulation of tumor cell growth and metastasis [70].

Recently, also the low-density receptor-related protein (LRP) has been shown to be cleaved by a *γ*-secretase-like activity [97]. It is important to note that LRPs receptors are activated by apolipoprotein E, a well-known risk factor for the developing of late onset AD in carriers of the *ε*_4_ alleles [98, 99]. It is, however, still unknown if the processing by *γ*-secretase and the apolipoprotein E-mediated signaling on neuronal LRPs might modulate a single pathway, and which is the physiological significance for these processes.

PS1 also modulates basal level of ERK1/2 activity through a ras-Raf-MEK-dependent pathway activated by a direct binding with the SH2 domain of Grb2 (Figure 2) [100–102]. ERK family is one of the most ubiquitous cellular signaling mechanisms, whose activation links extracellular stimuli to cell proliferation, survival, and differentiation, but also cell death and apoptosis [103–105]. In this respect, it is worth of note to observe, as mentioned above, that ERK1/2 pathway is also modulated by A*β*PP (Figures 1 and 2).

Taken together, these data suggest that PS1 and/or A*β*PP are able to modulate different intracellular signalling pathway through a plethora of intracellular mediators; when the signaling activated by PS1 and A*β*PP become dysfunctional in neurons, in particular the activation of the cell cycle-machinery induced by ERK1/2, the neurodegenerative process may be activated (Figure 2).

## 4. A*β*PP, Presenilins, and Cell Cycle

The hypothesis that cell-cycle abnormalities and aberrant neuron cell-cycle reentering may cause neuronal death in AD is supported by different experimental findings including AD patients brain analysis and data obtained by *in vitro *experiments.

Chromosome missegregation and trisomy 21 mosaicism have been associated with mutations in A*β*PP and PSs [72]. Aberrant expression of cell-cycle proteins and tetraploidy in neurons from AD patients have been described [106]. In AD brains, the activation of several cell-cycle components has been detected, including cdc2, cdk4, p16, Ki-67, cyclin B1 and cyclin D, p25 (the regulatory subunit of cdk5) [107, 108], as well as the increased expression activity of genes encoding for cell-cycle proteins [109]. It was observed that hippocampal pyramidal and basal forebrain neurons, in AD brain show markers of DNA replication [110], and it was speculated that the state of tetraploidy is lethal to neurons [110].

Increasing observations suggest that aberrant activation of cell cycle may affect the formation of neurofibrillary tangles with hyperphosphorylation of Tau protein in AD brain. It is well known that p25/cdk5 complex hyperphosphorylates Tau and reduces its ability to associate with microtubules [107]. On the other hand, cell-cycle activation can lead to apoptosis [108], and several studies showed the activation of caspases in AD brain [111–113]. Finally, cell-cycle defects represent a major neuropathological feature also in transgenic animal models of AD [108, 110, 114, 115].

As previously discussed [73], A*β*PP regulates ERK1/2 levels, its phosphorylation/translocation to the centrosome, and cell proliferation rate.

Additionally, in the same study, we showed that also PS1 interacts with Grb2 in the centrosomes and modulates ERK1/2 signaling. Thus, the proposed hypothesis is that both A*β*PP and PS1 participate in the same signaling pathway through Grb2 binding. Since many regulatory molecules are found at centrosomes, it was postulated that centrosomes might serve as signaling machinery modulating different cell functions [98]. In particular, since Grb2, A*β*PP, PS1, and pERK1/2 are all detectable in mitotic centrosomes, it is conceivable that these structures might anchor signal transduction pathways, integrate signals, and facilitate its conversion, into cellular functions (Figure 2).

In this scenario, it was proposed that PS1 and A*β*PP may determine the activation of ERK1/2 that, in turn, was responsible for the initiation of the cell cycle [73]; when these events occur in postmitotic neurons, the impossibility to complete cell division leads inevitably to neuronal apoptosis.

## 5. A*β*PP, Presenilins, and LRPs

Low-density lipoprotein receptors (LDLRs) are type I integral membrane proteins currently composed of 10 members. LDLR possesses a wide array of ligands with different functions from cellular cholesterol uptake in the liver to cell specification and neuronal positioning during embryogenesis. ApoE, complexed in HDL and VLDL, is the major ligand for these receptors, and, being the *ε*_4_ allele of APOE gene, the most relevant risk for the development of late-onset AD, several studies support a role for these receptors in the pathogenesis of AD [116]. Although the molecular mechanisms underlying the association between ApoE alleles and AD development have not yet been completely elucidated, ApoE, along with its receptor-LDLR and LDL-receptors related protein (LRP), was reported to modulate A*β* production and clearance. Lack of LDLRs increased amyloid deposition and impaired cognitive behavior in AD transgenic mice [117]. ApoE colocalizes in amyloid deposits in brain parenchyma [118], and its lipidation state affects the ability to bind A*β* [119].

Beside its role as A*β* chaperone, ApoE might modulate specific internalization and signaling events via binding to its receptors. Some of them possess shared adaptors with A*β*PP; in particular Fe65 and JIP1 bind to LRP8, LRP1, and megalin. Indeed *γ*-secretase cleavage regulates the intramembrane proteolysis of LRP8, LRP1, and of SOR-1/LRP11. It is tempting to speculate that LRPs could affect A*β*PP processing and signaling (and vice versa) through *γ*-secretase and ApoE-mediated stimuli.

LRPs possess at least one NPxY motif in their cytoplasmic tail (except SOR-1/LRP11), and this motif, present in A*β*PP as well, is critically required for receptor interaction with adaptors proteins and for internalization. It has been recently demonstrated that several LRPs family members modulate A*β*PP processing by affecting different aspects of A*β*PP trafficking [120]. For example, LRP8 is a member of the LDLR family that is highly expressed in the brain [121]. It has been recently proposed that the physiological role of LRP8 might include the regulation of signal transduction pathways rather than endocytosis of lipoproteins and other ligands [116]. It is known that LRP8 interacts with A*β*PP, enhancing the level of A*β*PP at the cell surface, and reducing its internalization [122, 123]. Overexpression of LRP8 induces an increase in A*β*PP association with lipid rafts and decreases A*β*PP-CTFs levels [116].

ApoE was reported to induce Dab1 phosphorylation and ERK1/2 activation and JNK inhibition *via* LRPs. This pathway depends on the presence of Ca^++^ influx through the NMDA receptor, but it is independent of Dab1 [124].

Overall these data indicate a likely involvement of LRP8 as modulator of A*β*PP processing, by affecting its endocytic trafficking and the proportion of A*β*PP present in lipid rafts. These events may have consequence on the *γ*-secretase-mediated cleavage of A*β*PP and on its neurodegeneration-related signaling activity.

Upon binding, LRP8 transduces reelin signaling during neuronal development [125], and recent evidence has indicated that it interacts with the NR2A and NR2B subunits of NMDA receptor [126], being involved in neuronal functions such as maturation of NMDA receptor composition in the hippocampus, and the regulation of long-term potentiation [127]. Importantly, it has been determined that LRP8 ligand reelin is found in neuritic plaques of transgenic mice overexpressing A*β*PP [128], suggesting a possible association with AD. Subsequently, a novel interaction between reelin and A*β*PP was discovered, leading to increase in the cell surface levels of A*β*PP and affecting A*β*PP processing and A*β* production [8]. It was shown that reelin signaling in excitatory synapses can restore normal synaptic plasticity, which is impaired by oligomeric A*β* peptides at concentrations within the range detectable in the brains of AD patients. At high concentrations of A*β* peptides, reelin can no longer overcome the A*β*-induced functional suppression, and this condition coincides with a complete blockade of the reelin-dependent phosphorylation of NR2 subunits in NMDA receptors. This reversal requires the LRP receptor-dependent activation of tyrosine kinases of the Src family. It was proposed a model in which A*β*, reelin, and LRP receptors modulate neurotransmission and thus synaptic stability as opposing regulators of synaptic gain [12]. A schematic representation of potential roles of LRPs in normal brain function and in neurodegenerative processes is depicted in Figure 3.

## 6. Small Nuclear RNA in AD

Recent discoveries in molecular genetics of mammalian genome have shed light on a widespread transcription of noncoding regions, devoted to the regulation of the protein-coding genome expression. The mechanisms of action of these transcripts are various and different in nature, although all of them are devoted to the regulation of fundamental genetic pathways involved in the determination of the cell phenotype [129–132].

Alternative splicing is a central component of human brain complexity whose regulatory mechanisms are still largely unclear. The recent discovery of factors that control alternative splicing might contribute to clarify the molecular basis of physiological and pathological processes [133]. In two recent works, we described the discovery of two novel RNA polymerase III-dependent, noncoding RNAs (ncRNA) transcripts, named 17A and 38A. In particular, it was shown that the expression of 17A induces an alternative splicing of GABA-B2 receptor leading to the formation of a nonfunctional protein. The ncRNA 17A is normally expressed in the human brain but is highly upregulated in the brain of AD patients. The stable expression of 17A in SH-SY5Y neuroblastoma cells enhances the secretion of A*β* and the A*β* x-42/A*β* x-40 peptide ratio. Indeed the synthesis of 17A is upregulated in response to inflammatory stimuli, suggesting that it may be induced by AD-related inflammation and that it could contribute to neurodegeneration in AD [134].

In the other study, we found that IL1-*α*-dependent up-regulation of another ncRNA, named 38A, drives the synthesis of an alternatively spliced form of the potassium channel-interacting protein (KCNIP4). The alternative KCNIP4 isoform cannot interact with the *γ*-secretase complex, resulting in modification of *γ*-secretase activity, A*β*PP processing, and increased secretion of *β*-amyloid enriched in the more toxic A*β* x-42 species.

This alternative splicing shift is observed at high frequency in tissue from AD patients, suggesting that RNA polymerase III transcribed ncRNA may be upstream determinants of alternative splicing of significantly proteins involved in the brain homeostasis and that their inflammation-dependent overexpression may induce alterations in the A*β* production contributing to the neurodegeneration during AD development [135].

In this context, a more detailed investigation of ncRNA functional mechanisms might allow to identify new molecular connections with neurodegenerative diseases like those identified in AD.

## 7. Environmental Factors and AD Pathoetiology

In recent years, several data have showed evidence that environmental and/or nutritional factors may play a causal, disruptive, and/or protective role in the development of AD although the initiating molecular events are not entirely known. While a direct causal role for aluminum or other transition metals (copper, zinc, and iron) in AD has not yet been definitively demonstrated, epidemiological evidence suggests that elevated levels of these metals in the brain may be linked to the development or progression of the neurodegenerative processes during AD [136].

Aluminum role in AD has been investigated for decades. Recent studies have identified aluminum in early neurofibrillary tangle (NFT) of hippocampal CA1 neurons from brains of aged patients [137]. However, aluminum contribution to AD remains controversial, lacking physiological mechanistic role.

Iron deposition in the brain is another important proposed mechanisms in the pathophysiology AD. Excessive iron can contribute to the formation of free radicals, leading to lipid peroxidation and neurotoxicity, which can result in cell membrane damage and cell death [138]. Recently, it has been shown that iron concentration in AD patients brain was significantly higher than those of nondemented controls. In particular iron deposition in parietal cortex and hippocampus at the early stage of AD were positively correlated with the severity of patients cognitive impairment [139].

Also zinc was reported to accelerate the aggregation of the A*β* peptides and to play a role in the control of inflammatory responses. Inflammation clearly occurs in pathologically vulnerable regions of the AD brain with increased expression of acute phase proteins and proinflammatory cytokines which are hardly evident in normal brain and that could participate in the induction of neuronal death. In particular, cytokine expression may be regulated by zinc availability, so influencing inflammatory network phenotypic expression [140].

New lines of study show that lead exposures in early life has been implicated in subsequent progression of amyloidogenesis in rodents during old age. This exposure resulted in an increase in proteins associated with AD pathology: A*β*PP and A*β* peptide [141].

Recent work has shown that *in vitro* metal ligands such as clioquinol (CQ) increase the intracellular level of copper. The increase in intracellular copper was correlated with a dramatic and rapid decrease in levels of extracellular A*β* including A*β*1–40 and 1–42 [142]. It has been previously reported that CQ/copper complexes trigger the activation of PI3K and its downstream modulator Akt and the inhibition of glycogen synthase kinase 3 that in turn potentiated ERK1/2 phosphorylation [143, 144].

It is not clear if and how environmental factors take part to pathway discussed in this review, in which both A*β*PP and PS1 participate in the same signaling pathway leading, through Grb2 binding, to ERK1/2 activation and neurodegeneration. However, we may speculate that ERK1/2 activation by copper may contribute to the signal transduction system activated by A*β*PP, and PSs.

## 8. Concluding Remarks

The toxicity of A*β* peptides, eventually triggered or modulated by environmental or genetic factors, is a central dogma in AD genesis, which has been recently challenged by new achievements [48]. In particular, A*β*PP and PS1 participate in a plethora of protein-protein interactions and signaling pathways, suggesting that beside their implication for amyloid formation might also modulate specific cell signaling events involved in neuronal homeostasis that in a pathological context may lead to neurodegeneration.

In this scenario, it is under investigation the possible contribution of other receptors, such as LRPs, which interact with A*β*PP, could modulate its processing, are often target of *γ*-secretase cleavage, and share with A*β*PP relevant adaptors such as Fe65 and JIP1. It is tempting to hypothesize that the role of ApoE isoform 4 in AD, rather than being linked only to A*β* formation and clearing, might be also due to a specific receptor-mediated function which hampers A*β*PP physiological signaling and homeostatic control. In this vision, a unique pathway in which ApoE isoform 4, LRPs, A*β*PP, and PSs share common signal transduction events may represent the keystone that may explain A*β* formation and neurodegeneration. 

We would like to underline that, among these events, A*β*PP and PSs may affect ERK1/2 signaling through ShcA/Grb2 transduction system, with a net relevance for cell-cycle regulation that in postmitotic neurons may lead to cell death. Also some LRPs, as possible modulators of A*β*PP processing by affecting its endocytic trafficking and the proportion of A*β*PP present in lipid rafts, as well as the activity of the *γ*-secretase complex, could modulate ERK1/2 signaling via ShcA/Grb2 or through parallel pathways.

A parallel and complementary issue is given by the brain complexity and by the largely unexplored world of noncoding genome. The tip of the iceberg hides potentially relevant genomic control systems that may explain the widespread phenotypic variability, even among familial patients, observed in AD.

A more deep understanding of these complexes mechanism is necessary, in order to open new prospects for therapeutic applications in neurodegenerative disorders.

## References

[1] Golde TE, Eckman CB (2003). Physiologic and pathologic events mediated by intramembranous and juxtamembranous proteolysis. *Science’s STKE*.

[2] Selkoe DJ, Podlisny MB (2002). Deciphering the genetic basis of Alzheimer’s disease. *Annual Review of Genomics and Human Genetics*.

[3] Hardy J, Selkoe DJ (2002). The amyloid hypothesis of Alzheimer’s disease: progress and problems on the road to therapeutics. *Science*.

[4] Selkoe DJ, Wolfe MS (2007). Presenilin: running with scissors in the membrane. *Cell*.

[5] Wolfe MS (2007). When loss is gain: reduced presenilin proteolytic function leads to increased A*β*42/A*β*40. Talking point on the role of presenilin mutations in Alzheimer disease. *EMBO Reports*.

[6] Heilig EA, Xia W, Shen J, Kelleher RJ (2010). A presenilin-1 mutation identified in familial Alzheimer disease with cotton wool plaques causes a nearly complete loss of *γ*-secretase activity. *Journal of Biological Chemistry*.

[7] Shen J, Kelleher RJ (2007). The presenilin hypothesis of Alzheimer’s disease: evidence for a loss-of-function pathogenic mechanism. *Proceedings of the National Academy of Sciences of the United States of America*.

[8] Hoe HS, Fu Z, Makarova A (2009). The effects of amyloid precursor protein on postsynaptic composition and activity. *Journal of Biological Chemistry*.

[9] da Cruz e Silva EF, da Cruz e Silva OA (2003). Protein phosphorylation and APP metabolism. *Neurochemical Research*.

[10] De Strooper B, Annaert W (2000). Proteolytic processing and cell biological functions of the amyloid precursor protein. *Journal of Cell Science*.

[11] Bu G (2009). Apolipoprotein e and its receptors in Alzheimer’s disease: pathways, pathogenesis and therapy. *Nature Reviews Neuroscience*.

[12] Durakoglugil MS, Chen Y, White CL, Kavalali ET, Herz J (2009). Reelin signaling antagonizes *β*-amyloid at the synapse. *Proceedings of the National Academy of Sciences of the United States of America*.

[13] Wagner T, Pietrzik CU The role of lipoprotein receptors on the physiological function of APP.

[14] Golde TE (2003). Alzheimer disease therapy: can the amyloid cascade be halted?. *Journal of Clinical Investigation*.

[15] Levy-Lahad E, Wasco W, Poorkaj P (1995). Candidate gene for the chromosome 1 familial Alzheimer’s disease locus. *Science*.

[16] Rogaev EI, Sherrington R, Rogaeva EA (1995). Familial Alzheimer’s disease in kindreds with missense mutations in a gene on chromosome 1 related to the Alzheimer’s disease type 3 gene. *Nature*.

[17] Bertram L, Hiltunen M, Parkinson M (2005). Family-based association between alzheimer’s disease and variants in UBQLN1. *The New England Journal of Medicine*.

[18] Bertram L, Hsiao M, McQueen MB (2007). The LDLR locus in alzheimer’s disease: a family-based study and meta-analysis of case-control data. *Neurobiology of Aging*.

[19] Bertram L, Hsiao M, Mullin K (2005). ACAT1 is not associated with Alzheimer’s disease in two independent family-based samples. *Molecular Psychiatry*.

[20] Bertram L, Parkinson M, McQueen MB (2005). Further evidence for LBP-1c/CP2/LSF association in Alzheimer’s disease families. *Journal of Medical Genetics*.

[21] Bertram L, Tanzi RE (2005). The genetic epidemiology of neurodegenerative disease. *Journal of Clinical Investigation*.

[22] Dickerson BC, Salat DH, Greve DN (2005). Increased hippocampal activation in mild cognitive impairment compared to normal aging and AD. *Neurology*.

[23] Tanzi RE, Bertram L (2005). Twenty years of the Alzheimer’s disease amyloid hypothesis: a genetic perspective. *Cell*.

[24] Zambrano N, Buxbaum JD, Minopoli G (1997). Interaction of the phosphotyrosine interaction/phosphotyrosine binding-related domains of Fe65 with wild-type and mutant Alzheimer’s *β*-amyloid precursor proteins. *Journal of Biological Chemistry*.

[25] Neve RL, McPhie DL (2006). The cell cycle as a therapeutic target for Alzheimer’s disease. *Pharmacology and Therapeutics*.

[26] Ando K, Iijima KI, Elliott JI, Kirino Y, Suzuki T (2001). Phosphorylation-dependent regulation of the interaction of amyloid precursor protein with Fe65 affects the production of *β*-amyloid. *Journal of Biological Chemistry*.

[27] Cao X, Sudhof TC (2001). A transcriptively active complex of APP with Fe65 and histone acetyltransferase Tip60. *Science*.

[28] Cao X, Sudhof TC (2004). Dissection of amyloid-*β* precursor protein-dependent transcriptional transactivation. *Journal of Biological Chemistry*.

[29] Kinoshita A, Whelan CM, Smith CJ, Berezovska O, Hyman BT (2002). Direct visualization of the gamma secretase-generated carboxyl-terminal domain of the amyloid precursor protein: association with Fe65 and translocation to the nucleus. *Journal of Neurochemistry*.

[30] Hebert SS, Serneels L, Tolia A (2006). Regulated intramembrane proteolysis of amyloid precursor protein and regulation of expression of putative target genes. *EMBO Reports*.

[31] Hass MR, Yankner BA (2005). A *γ*-secretase-independent mechanism of signal transduction by the amyloid precursor protein. *Journal of Biological Chemistry*.

[32] Stante M, Minopoli G, Passaro F, Raia M, Vecchio LD, Russo T (2009). Fe65 is required for Tip60-directed histone H4 acetylation at DNA strand breaks. *Proceedings of the National Academy of Sciences of the United States of America*.

[33] Ma QH, Futagawa T, Yang WL (2008). A TAG1-APP signalling pathway through Fe65 negatively modulates neurogenesis. *Nature Cell Biology*.

[34] Vogt DL, Thomas D, Galvan V, Bredesen DE, Lamb BT, Pimplikar SW (2009). Abnormal neuronal networks and seizure susceptibility in mice overexpressing the APP intracellular domain. *Neurobiology of Aging*.

[35] Belyaev ND, Nalivaeva NN, Makova NZ, Turner AJ (2009). Neprilysin gene expression requires binding of the amyloid precursor protein intracellular domain to its promoter: implications for Alzheimer disease. *EMBO Reports*.

[36] Tarr PE, Roncarati R, Pelicci G, Pelicci PG, D’Adamio L (2002). Tyrosine phosphorylation of the *β*-amyloid precursor protein cytoplasmic tail promotes interaction with Shc. *Journal of Biological Chemistry*.

[37] Borg JP, Ooi J, Levy E, Margolis B (1996). The phosphotyrosine interaction domains of X11 and FE65 bind to distinct sites on the YENPTY motif of amyloid precursor protein. *Molecular and Cellular Biology*.

[38] Fiore F, Zambrano N, Minopoli G, Donini V, Duilio A, Russo T (1995). The regions of the Fe65 protein homologous to the phosphotyrosine interaction/phosphotyrosine binding domain of Shc bind the intracellular domain of the Alzheimer’s amyloid precursor protein. *Journal of Biological Chemistry*.

[39] Howell BW, Lanier LM, Frank R, Gertler FB, Cooper JA (1999). The disabled 1 phosphotyrosine-binding domain binds to the internalization signals of transmembrane glycoproteins and to phospholipids. *Molecular and Cellular Biology*.

[40] Zambrano N, Bruni P, Minopoli G (2001). The *β*-amyloid precursor protein APP is tyrosine-phosphorylated in cells expressing a constitutively active form of the Abl protoncogene. *Journal of Biological Chemistry*.

[41] Scheinfeld MH, Roncarati R, Vito P, Lopez PA, Abdallah M, D’Adamio L (2002). Jun NH2-terminal kinase (JNK) interacting protein 1 (JIP1) binds the cytoplasmic domain of the Alzheimer’s *β*-amyloid precursor protein (APP). *Journal of Biological Chemistry*.

[42] Sabo SL, Ikin AF, Buxbaum JD, Greengard P (2001). The Alzheimer amyloid precursor protein (APP) and FE65, an APP-binding protein, regulate cell movement. *Journal of Cell Biology*.

[43] Howell BW, Hawkes R, Soriano P, Cooper JA (1997). Neuronal position in the developing brain is regulated by mouse disabled-1. *Nature*.

[44] Parisiadou L, Efthimiopoulos S (2007). Expression of mDab1 promotes the stability and processing of amyloid precursor protein and this effect is counteracted by X11*α*. *Neurobiology of Aging*.

[45] Borg JP, Yang Y, De Taddeo-Borg M, Margolis B, Turner RS (1998). The X11*α* protein slows cellular amyloid precursor protein processing and reduces a*β*40 and a*β*42 secretion. *Journal of Biological Chemistry*.

[46] Rogelj B, Mitchell JC, Miller CC, McLoughlin DM (2006). The X11/Mint family of adaptor proteins. *Brain Research Reviews*.

[47] Taru H, Kirino Y, Suzuki T (2002). Differential roles of JIP scaffold proteins in the modulation of amyloid precursor protein metabolism. *Journal of Biological Chemistry*.

[48] Hardy J (2009). The amyloid hypothesis for Alzheimer’s disease: a critical reappraisal. *Journal of Neurochemistry*.

[49] Zhou D, Noviello C, D’Ambrosio C, Scaloni A, D’Adamio L (2004). Growth factor receptor-bound protein 2 interaction with the tyrosine-phosphorylated tail of amyloid *β* precursor protein is mediated by its Src homology 2 domain. *Journal of Biological Chemistry*.

[50] Cattaneo E, Pelicci PG (1998). Emerging roles for SH2/PTB-containing Shc adaptor proteins in the developing mammalian brain. *Trends in Neurosciences*.

[51] Luzi L, Confalonieri S, Di Fiore PP, Pelicci PG (2000). Evolution of Shc functions from nematode to human. *Current Opinion in Genetics and Development*.

[52] Dankort D, Maslikowski B, Warner N (2001). Grb2 and Shc adapter proteins play distinct roles in Neu (ErbB-2)-induced mammary tumorigenesis: implications for human breast cancer. *Molecular and Cellular Biology*.

[53] Puto LA, Pestonjamasp K, King CC, Bokoch GM (2003). p21-activated kinase 1 (PAK1) interacts with the Grb2 adapter protein to couple to growth factor signaling. *Journal of Biological Chemistry*.

[54] Venezia V, Nizzari M, Repetto E (2006). Amyloid precursor protein modulates ERK-1 and -2 signaling. *Annals of the New York Academy of Sciences*.

[55] Russo C, Dolcini V, Salis S (2002). Signal transduction through tyrosine-phosphorylated C-terminal fragments of amyloid precursor protein via an enhanced interaction with Shc/Grb2 adaptor proteins in reactive astrocytes of Alzheimer’s disease brain. *Journal of Biological Chemistry*.

[56] Russo C, Venezia V, Salis S, Dolcini V, Schettini G (2002). Molecular aspects of neurodegeneration in Alzheimer’s disease. *Functional Neurology*.

[57] Russo C, Violani E, Salis S (2002). Pyroglutamate-modified amyloid *β*-peptides—a*β*N3(pE)—strongly affect cultured neuron and astrocyte survival. *Journal of Neurochemistry*.

[58] Roder HM, Eden PA, Ingram VM (1993). Brain protein kinase PK40(erk) converts TAU into a PHF-like form as found in Alzheimer’s disease. *Biochemical and Biophysical Research Communications*.

[59] Guise S, Braguer D, Carles G, Delacourte A, Briand C (2001). Hyperphosphorylation of tau is mediated by erk activation during anticancer drug-induced apoptosis in neuroblastoma cells. *Journal of Neuroscience Research*.

[60] Brouillet E, Trembleau A, Galanaud D (1999). The amyloid precursor protein interacts with G(o) heterotrimeric protein within a cell compartment specialized in signal transduction. *Journal of Neuroscience*.

[61] Edmonds BT, Moomaw CR, Hsu JT, Slaughter C, Ellis L (1990). The p38 and p34 polypeptides of growth cone particle membranes are the *α*- and *β*-subunits of G proteins. *Developmental Brain Research*.

[62] Giambarella U, Yamatsuji T, Okamoto T (1997). G protein *βγ* complex-mediated apoptosis by familial Alzheimer’s disease mutant of APP. *The EMBO Journal*.

[63] Nishimoto I, Okamoto T, Matsuura Y (1993). Alzheimer amyloid protein precursor complexes with brain GTP-binding protein G(o). *Nature*.

[64] Chow N, Korenberg JR, Chen XN, Neve RL (1996). APP-BP1, a novel protein that binds to the carboxyl-terminal region of the amyloid precursor protein. *Journal of Biological Chemistry*.

[65] McPhie DL, Coopersmith R, Hines-Peralta A (2003). DNA synthesis and neuronal apoptosis caused by familial Alzheimer disease mutants of the amyloid precursor protein are mediated by the p21 activated kinase PAK3. *Journal of Neuroscience*.

[66] Nizzari M, Venezia V, Bianchini P (2007). Amyloid precursor protein and presenilin 1 interaction studied by FRET in human H4 cells. *Annals of the New York Academy of Sciences*.

[67] Venezia V, Nizzari M, Carlo P, Corsaro A, Florio T, Russo C (2007). Amyloid precursor protein and presenilin involvement in cell signaling. *Neurodegenerative Diseases*.

[68] Venezia V, Russo C, Repetto E (2004). Apoptotic cell death and amyloid precursor protein signaling in neuroblastoma SH-SY5Y cells. *Annals of the New York Academy of Sciences*.

[69] De Strooper B (2003). Aph-1, Pen-2, and nicastrin with presenilin generate an active *γ*-secretase complex. *Neuron*.

[70] Murakami D, Okamoto I, Nagano O (2003). Presenilin-dependent *γ*-secretase activity mediates the intramembranous cleavage of CD44. *Oncogene*.

[71] Selkoe DJ (2001). Presenilin, notch, and the genesis and treatment of Alzheimer’s disease. *Proceedings of the National Academy of Sciences of the United States of America*.

[72] Li J, Xu M, Zhou H, Ma J, Potter H (1997). Alzheimer presenilins in the nuclear membrane, interphase kinetochores, and centrosomes suggest a role in chromosome segregation. *Cell*.

[73] Nizzari M, Venezia V, Repetto E (2007). Amyloid precursor protein and presenilin1 interact with the adaptor GRB2 and modulate ERK1,2 signaling. *Journal of Biological Chemistry*.

[74] Gandy S, Naslund J, Nordstedt C (2001). Alzheimer’s disease: molecular consequences of presenilin-1 mutation. *Nature*.

[75] Russo C, Schettini G, Saido TC (2000). Presenilin-1 mutations in Alzheimer’s disease. *Nature*.

[76] Ezquerra M, Lleo A, Castellvi M (2003). A novel mutation in the PSEN2 gene (T430M) associated with variable expression in a family with early-onset Alzheimer disease. *Archives of Neurology*.

[77] Baulac S, LaVoie MJ, Kimberly WT (2003). Functional *γ*-secretase complex assembly in Golgi/trans-Golgi network: interactions among presenilin, nicastrin, Aph1, Pen-2, and *γ*-secretase substrates. *Neurobiology of Disease*.

[78] Kopan R, Goate A (2000). A common enzyme connects Notch signaling and Alzheimer’s disease. *Genes and Development*.

[79] Schroeter EH, Kisslinger JA, Kopan R (1998). Notch-1 signalling requires ligand-induced proteolytic release of intracellular domain. *Nature*.

[80] Dale TC (1998). Signal transduction by the Wnt family of ligands. *Biochemical Journal*.

[81] Spink KE, Polakis P, Weis WI (2000). Structural basis of the Axin-adenomatous polyposis coli interaction. *The EMBO Journal*.

[82] Marambaud P, Shioi J, Serban G (2002). A presenilin-1/*γ*-secretase cleavage releases the E-cadherin intracellular domain and regulates disassembly of adherens junctions. *The EMBO Journal*.

[83] Marambaud P, Wen PH, Dutt A (2003). A CBP binding transcriptional repressor produced by the PS1/ *ε*-cleavage of N-Cadherin is inhibited by PS1 FAD mutations. *Cell*.

[84] Zhang Z, Hartmann H, Do VM (1998). Destabilization of *β*-catenin by mutations in presenilin-1 potentiates neuronal apoptosis. *Nature*.

[85] Phiel CJ, Wilson CA, Lee VM, Klein PS (2003). GSK-3*α* regulates production of Alzheimer’s disease amyloid-*β* peptides. *Nature*.

[86] Ghosal K, Vogt DL, Liang M, Shen Y, Lamb BT, Pimplikar SW (2009). Alzheimer’s disease-like pathological features in transgenic mice expressing the APP intracellular domain. *Proceedings of the National Academy of Sciences of the United States of America*.

[87] Xia X, Qian S, Soriano S (2001). Loss of presenilin 1 is associated with enhanced *β*-catenin signaling and skin tumorigenesis. *Proceedings of the National Academy of Sciences of the United States of America*.

[88] Artavanis-Tsakonas S, Rand MD, Lake RJ (1999). Notch signaling: cell fate control and signal integration in development. *Science*.

[89] Osborne B, Miele L (1999). Notch and the immune system. *Immunity*.

[90] Thinakaran G, Parent AT (2004). Identification of the role of presenilins beyond Alzheimer’s disease. *Pharmacological Research*.

[91] Saura CA, Choi SY, Beglopoulos V (2004). Loss of presenilin function causes impairments of memory and synaptic plasticity followed by age-dependent neurodegeneration. *Neuron*.

[92] Albericit A, Moratto D, Benussi L (1999). Presenilin 1 protein directly interacts with Bcl-2. *Journal of Biological Chemistry*.

[93] Passer BJ, Pellegrini L, Vito P, Ganjei JK, D’Adamio L (1999). Interaction of Alzheimer’s presenilin-1 and presenilin-2 with Bcl-X(L). A potential role in modulating the threshold of cell death. *Journal of Biological Chemistry*.

[94] Gumbiner BM (2000). Regulation of cadherin adhesive activity. *Journal of Cell Biology*.

[95] Yagi T, Takeichi M (2000). Cadherin superfamily genes: functions, genomic organization, and neurologic diversity. *Genes and Development*.

[96] Uemura K, Kitagawa N, Kohno R (2003). Presenilin 1 is involved in maturation and trafficking of N-cadherin to the plasma membrane. *Journal of Neuroscience Research*.

[97] May P, Reddy YK, Herz J (2002). Proteolytic processing of low density lipoprotein receptor-related protein mediates regulated release of its intracellular domain. *Journal of Biological Chemistry*.

[98] Corder EH, Saunders AM, Strittmatter WJ (1993). Gene dose of apolipoprotein E type 4 allele and the risk of Alzheimer’s disease in late onset families. *Science*.

[99] Strittmatter WJ, Saunders AM, Schmechel D (1993). Apolipoprotein E: high-avidity binding to *β*-amyloid and increased frequency of type 4 allele in late-onset familial Alzheimer disease. *Proceedings of the National Academy of Sciences of the United States of America*.

[100] Kang DE, Yoon IS, Repetto E (2005). Presenilins mediate phosphatidylinositol 3-kinase/AKT and ERK activation via select signaling receptors: selectivity of PS2 in platelet-derived growth factor signaling. *Journal of Biological Chemistry*.

[101] Kim HK, Jeong MJ, Kong MY (2005). Inhibition of Shc/Grb2 protein-protein interaction suppresses growth of B104-1-1 tumors xenografted in nude mice. *Life Sciences*.

[102] Kim MY, Park JH, Choi EJ, Park HS (2005). Presenilin acts as a positive regulator of basal level activity of ERK through the Raf-MEK1 signaling pathway. *Biochemical and Biophysical Research Communications*.

[103] Johnson GL, Lapadat R (2002). Mitogen-activated protein kinase pathways mediated by ERK, JNK, and p38 protein kinases. *Science*.

[104] Kyriakis JM (1999). Making the connection: coupling of stress-activated ERK/MAPK (extracellular-signal-regulated kinase/mitogen-activated protein kinase) core signalling modules to extracellular stimuli and biological responses. *Biochemical Society Symposium*.

[105] Sawe N, Steinberg G, Zhao H (2008). Dual roles of the MAPK/ERK1/2 cell signaling pathway after stroke. *Journal of Neuroscience Research*.

[106] Lee EY, Chang CY, Hu N (1992). Mice deficient for Rb are nonviable and show defects in neurogenesis and haematopoiesis. *Nature*.

[107] Patrick GN, Zukerberg L, Nikolic M, de La Monte S, Dikkes P, Tsai LH (1999). Conversion of p35 to p25 deregulates Cdk5 activity and promotes neurodegeneration. *Nature*.

[108] Vincent I, Rosado M, Davies P (1996). Mitotic mechanisms in Alzheimer’s disease?. *Journal of Cell Biology*.

[109] Chow N, Cox C, Callahan LM, Weimer JM, Guo L, Coleman PD (1998). Expression profiles of multiple genes in single neurons of Alzheimer’s disease. *Proceedings of the National Academy of Sciences of the United States of America*.

[110] Yang Y, Geldmacher DS, Herrup K (2001). DNA replication precedes neuronal cell death in Alzheimer’s disease. *Journal of Neuroscience*.

[111] Raina AK, Hochman A, Zhu X (2001). Abortive apoptosis in Alzheimer’s disease. *Acta Neuropathologica*.

[112] Selznick LA, Holtzman DM, Han BH (1999). In situ immunodetection of neuronal caspase-3 activation in Alzheimer disease. *Journal of Neuropathology and Experimental Neurology*.

[113] Stadelmann C, Deckwerth TL, Srinivasan A (1999). Activation of caspase-3 in single neurons and autophagic granules of granulovacuolar degeneration in Alzheimer’s disease: evidence for apoptotic cell death. *American Journal of Pathology*.

[114] Dranovsky A, Vincent I, Gregori L (2001). Cdc2 phosphorylation of nucleolin demarcates mitotic stages and Alzheimer’s disease pathology. *Neurobiology of Aging*.

[115] Yang Y, Varvel NH, Lamb BT, Herrup K (2006). Ectopic cell cycle events link human Alzheimer’s disease and amyloid precursor protein transgenic mouse models. *Journal of Neuroscience*.

[116] Marzolo MP, Bu G (2009). Lipoprotein receptors and cholesterol in APP trafficking and proteolytic processing, implications for Alzheimer’s disease. *Seminars in Cell and Developmental Biology*.

[117] Cao D, Fukuchi K, Wan H, Kim H, Li L (2006). Lack of LDL receptor aggravates learning deficits and amyloid deposits in Alzheimer transgenic mice. *Neurobiology of Aging*.

[118] Burns MP, Noble WJ, Olm V (2003). Co-localization of cholesterol, apolipoprotein E and fibrillar A*β* in amyloid plaques. *Molecular Brain Research*.

[119] Tokuda T, Calero M, Matsubara E (2000). Lipidation of apolipoprotein E influences its isoform-specific interaction with Alzheimer’s amyloid *β* peptides. *Biochemical Journal*.

[120] Ulery PG, Beers J, Mikhailenko I (2000). Modulation of *β*-amyloid precursor protein processing by the low density lipoprotein receptor-related protein (LRP). Evidence that LRP contributes to the pathogenesis of Alzheimer’s disease. *Journal of Biological Chemistry*.

[121] Kim DH, Iijima H, Goto K (1996). Human apolipoprotein E receptor 2: a novel lipoprotein receptor of the low density lipoprotein receptor family predominantly expressed in brain. *Journal of Biological Chemistry*.

[122] Fuentealba RA, Barria MI, Lee J (2007). ApoER2 expression increases abeta production while decreasing amyloid precursor protein (APP) endocytosis: possible role in the partitioning of APP into lipid rafts and in the regulation of gamma-secretase activity. *Molecular Neurodegeneration*.

[123] Hoe HS, Harris DC, Rebeck GW (2005). Multiple pathways of apolipoprotein E signaling in primary neurons. *Journal of Neurochemistry*.

[124] Hoe HS, Wessner D, Beffert U, Becker AG, Matsuoka Y, Rebeck GW (2005). F-spondin interaction with the apolipoprotein E receptor ApoEr2 affects processing of amyloid precursor protein. *Molecular and Cellular Biology*.

[125] Hoe HS, Kea JL, Carney RS (2009). Interaction of reelin with amyloid precursor protein promotes neurite outgrowth. *Journal of Neuroscience*.

[126] Hoe HS, Pocivavsek A, Chakraborty G (2006). Apolipoprotein E receptor 2 interactions with the N-Methyl-D-aspartate receptor. *Journal of Biological Chemistry*.

[127] Sinagra M, Verrier D, Frankova D (2005). Reelin, very-low-density lipoprotein receptor, and apolipoprotein E receptor 2 control somatic NMDA receptor composition during hippocampal maturation in vitro. *Journal of Neuroscience*.

[128] Botella-Lopez A, Burgaya F, Gavin R (2006). Reelin expression and glycosylation patterns are altered in Alzheimer’s disease. *Proceedings of the National Academy of Sciences of the United States of America*.

[129] Dahary D, Elroy-Stein O, Sorek R (2005). Naturally occurring antisense: transcriptional leakage or real overlap?. *Genome Research*.

[130] Mattick JS (2004). RNA regulation: a new genetics?. *Nature Reviews Genetics*.

[131] Reis EM, Nakaya HI, Louro R (2004). Antisense intronic non-coding RNA levels correlate to the degree of tumor differentiation in prostate cancer. *Oncogene*.

[132] Yelin R, Dahary D, Sorek R (2003). Widespread occurrence of antisense transcription in the human genome. *Nature Biotechnology*.

[133] Pagano A, Castelnuovo M, Tortelli F, Ferrari R, Dieci G, Cancedda R (2007). New small nuclear RNA gene-like transcriptional units as sources of regulatory transcripts. *Plos Genetics*.

[134] Massone S, Vassallo I, Fiorino G (2011). 17A, a novel non-coding RNA, regulates GABA B alternative splicing and signaling in response to inflammatory stimuli and in Alzheimer disease. *Neurobiology of Disease*.

[135] Massone S, Vassallo I, Castelnuovo M (2011). RNA polymerase III drives alternative splicing of the potassium channel-interacting protein contributing to brain complexity and neurodegeneration. *Journal of Cell Biology*.

[136] Shcherbatykh I, Carpenter DO (2007). The role of metals in the etiology of Alzheimer’s disease. *Journal of Alzheimer’s Disease*.

[137] Walton JR (2010). Evidence for participation of aluminum in neurofibrillary tangle formation and growth in Alzheimer’s disease. *Journal of Alzheimer’s Disease*.

[138] Casadesus G, Smith MA, Zhu X (2004). Alzheimer disease: evidence for a central pathogenic role of iron-mediated reactive oxygen species. *Journal of Alzheimer’s Disease*.

[139] Qin Y, Zhu W, Zhan C (2011). Investigation on positive correlation of increased brain iron deposition with cognitive impairment in Alzheimer disease by using quantitative MR **R**2**'** mapping. *Journal of Huazhong University of Science and Technology*.

[140] Vasto S, Candore G, Listi F (2008). Inflammation, genes and zinc in Alzheimer’s disease. *Brain Research Reviews*.

[141] Joshi S, Guleria RS, Pan J, DiPette D, Singh US (2007). Heterogeneity in retinoic acid signaling in neuroblastomas: role of matrix metalloproteinases in retinoic acid-induced differentiation. *Biochimica et Biophysica Acta*.

[142] Filiz G, Price KA, Caragounis A, Du T, Crouch PJ, White AR (2008). The role of metals in modulating metalloprotease activity in the AD brain. *European Biophysics Journal*.

[143] Caragounis A, Du T, Filiz G (2007). Differential modulation of Alzheimer’s disease amyloid *β*-peptide accumulation by diverse classes of metal ligands. *Biochemical Journal*.

[144] White AR, Du T, Laughton KM (2006). Degradation of the Alzheimer disease amyloid *β*-peptide by metal-dependent up-regulation of metalloprotease activity. *Journal of Biological Chemistry*.

